# Ⅰ、Ⅱ相药物代谢酶遗传多态性与晚期非小细胞肺癌化疗疗效的关系

**DOI:** 10.3779/j.issn.1009-3419.2011.11.05

**Published:** 2011-11-20

**Authors:** 伟英 李, 文涛 岳, 学惠 杨, 春彦 张, 玥 王

**Affiliations:** 101149 北京，北京市结核病胸部肿瘤研究所，细胞生物学研究室 Department of Cell Molecular Biology, Beijing Thoracic Tumour and Tuberculosis Research Institute, Beijing 101149, China

**Keywords:** 肺肿瘤, 药物代谢酶, 化疗效果, 预后, Lung neoplasms, Drugs metabolism enzymes, Chemotherapy response, Prognosis

## Abstract

**背景与目的:**

目前药物代谢酶遗传多态性与化疗疗效关系的研究结果多不一致，本研究旨在探讨细胞色素P4501A1（cytochrome P450 1A1, *CYP1A1*）、2E1（cytochrome P450 2E1, *CYP2E1*）、2D6（cytochrome P450 2D6, *CYP2D6*）和谷胱甘肽硫转移酶M1（glutathione S-transferase M1, *GSTM1*）基因多态性与晚期非小细胞肺癌化疗疗效以及与肺癌患者预后的关系。

**方法:**

采用PCR和PCR-RFLP技术对肺癌患者4种药物代谢酶基因分型，并对他们进行5年跟踪随访。

**结果:**

携带B型*CYP1A1*和缺陷性*GSTM1*肺癌患者比其它基因型患者化疗疗效好（*P* < 0.001）。携带A型*CYP1A1*肺癌患者接受非铂类化疗药物治疗比B型和C型患者疗效好（*P*=0.041）; 携带缺陷性*GSTM1*肺癌患者接受铂类化疗药物治疗疗效比功能型患者疗效好（*P*=0.011）。4种酶对晚期非小细胞肺癌患者总生存期（overall survival, OS）没有明显影响（*P* > 0.05）。

**结论:**

A型*CYP1A1*肺癌患者接受非铂类化疗药物治疗比B型和C型患者疗效好; 缺陷性*GSTM1*肺癌患者接受铂类化疗药物治疗比功能型患者疗效好。4种酶基因多态对晚期非小细胞肺癌患者OS影响没有明显统计学差异。

## Abstract (en)

**Background and objective:**

Currently available studies on the polymorphisms of drug-metabolizing enzymes and their chemotherapeutic effects in non-small cell lung cancer are not consistent. In the present study, the relationship of the gene polymorphisms of cytochrome P450 1A1 (*CYP1A1*), cytochrome P450 2E1 (*CYP2E1*), cytochrome P450 2D6 (*CYP2D6*), and glutathione S-transferase M1 (*GSTM1*) enzymes with chemotherapeutic effects were investigated. The effects of these relationships on the survival of advanced non-small cell lung cancer patients were also examined.

**Methods:**

Four drug metabolism enzymes were genotyped in lung cancer patients by polymerase chain reaction (PCR) and PCR-restriction fragment length polymorphism. These patients were followed for five years.

**Results:**

The chemotherapeutic effect on patients carrying B-type *CYP1A1* and null-type *GSTM1* was better than on those carrying other types (*P* < 0.001). The chemotherapeutic effect on patients carrying A-type *CYP1A1* was better than on those carrying the B and C types when non-platinum drugs were administered (*P*=0.041). The chemotherapeutic effect on patients carrying null-type *GSTM1* was better than on those carrying the functional type when platinum drugs were administered (*P*=0.011). The four enzymes did not affect the overall survival (OS) of advanced non-small cell lung cancer patients (*P* > 0.05).

**Conclusion:**

The chemotherapeutic effect on patients carrying A-type *CYP1A1* was better than on those carrying the B and C types when non-platinum drugs were administered. The chemotherapeutic effect on patients carrying null-type *GSTM1* was better than on those carrying the functional type when platinum drugs were administered. The four enzymes did not affect the OS of advanced non-small cell lung cancer patients.

肺癌是当今世界上发病率和死亡率极高的恶性肿瘤之一。肺癌中约有80%为非小细胞肺癌（non-small cell lung cancer, NSCLC），由于缺乏有效的早期诊断方法，确诊时肺癌多为晚期，因此化学药物治疗在NSCLC的治疗中占据主要地位，但对于不同的患者化疗效果差异很大。许多资料^[1, 2]^显示遗传因素对药物作用和毒性的影响显著，诸如编码药物代谢酶可以影响抗癌药物的动力学和药效参数。药物代谢酶催化两个阶段反应，大多数药物都要经过第一阶段Ⅰ相代谢酶反应解毒或者使无活性的前体药物活化，第二阶段Ⅱ相代谢酶通常结合第一阶段产物、其它反应介质或是生成更有利于肾脏或胆汁排泄的极性衍生物，这些酶的多态性可引发治疗药物不同的治疗药物动力学和药效的分布图，有研究^[3-5]^显示Ⅰ、Ⅱ相代谢酶功能型多态性可能影响肿瘤患者的临床预后。

为了进一步评估Ⅰ、Ⅱ相代谢酶功能型多态性与化疗疗效的关系以及对肺癌患者预后的影响，我们选择参与药物代谢第一阶段反应的谷胱甘肽硫转移酶M1（glutathione S-transferase M1, *GSTM1*）和第二阶段反应的细胞色素P4501A1（cytochrome P450 1A1, *CYP1A1*）、2E1（cytochrome P450 2E1, *CYP2E1*）、2D6（cytochrome P450 2D6, *CYP2D6*）作为研究对象，检测肺癌患者四种基因多态性频率分布，分析它们与吸烟、化疗疗效、化疗方案、病理分型等因素之间的关系以及与患者生存之间的关系。

## 1 材料与方法

### 1.1 临床资料

选取北京胸科医院2006年1月-2006年6月127例晚期NSCLC患者，所有患者均为细胞学或组织学确诊的肺癌，无其它部位肿瘤，既往未经放疗和化疗。收集研究对象的人口学资料、职业史、吸烟史、饮酒史等，部分资料见表 1。

**1:** 研究对象的一般资料 General characteristics of the subjects

	Response	No response	*P*
Gender			0.091
Male	43	37	
Female	33	14	
Age (yr)			0.305
Mean	56.7	54.8	
Range	22-82	24-78	
Histological type			0.130
Squamous cell carcinoma	16	14	
Adenocarcinoma	30	20	
Other	30	17	

### 1.2 DNA的提取

收集研究对象的外周血，用常规的酚-氯仿抽提法提取DNA，于-20 ℃保存。

### 1.3 基因分型

引物由上海生物工程有限公司合成，引物序列见表 2，基因分型判断见图 1-图 4。

**2:** CYP1A1、2E1、2D6和GSTM1基因分型引物序列 The primer sequences genotyping CYP1A1, 2E1, 2D6 and GSTM1

Gene	Sequences of primers
*GSTM1*	P1：5’–GAACTCCCTGAAAAGCTAAAGC–3’
	P2：5’–GTTGGGCTCAAATATACGGTGG–3’
	*β*1：5’–CAACTTCATCCACGTTCACC–3’
	*β*2：5’–GAAGAGCCAAGGACAGGTAC–3’
*CYP1A1*	P1：5’–TAGGAGTCTTGTCTCATGCCT–3’
	P2：5’–CAGTGAAGAGGTGTAGAAGCT–3’
*CYP2E1*	P1：5’–TTCATTCTGTCTTCTAACTGG–3’
	P2：5’–CCAGTCGAGTCTACATTGTCA–3’
*CYP2D6*	P1：5’–CCATTTGGTAGTGAGGCAGGTAT–3’
	P2：5’–CACCATCCATGTTTGCTTCTGGT–3’

**1:**
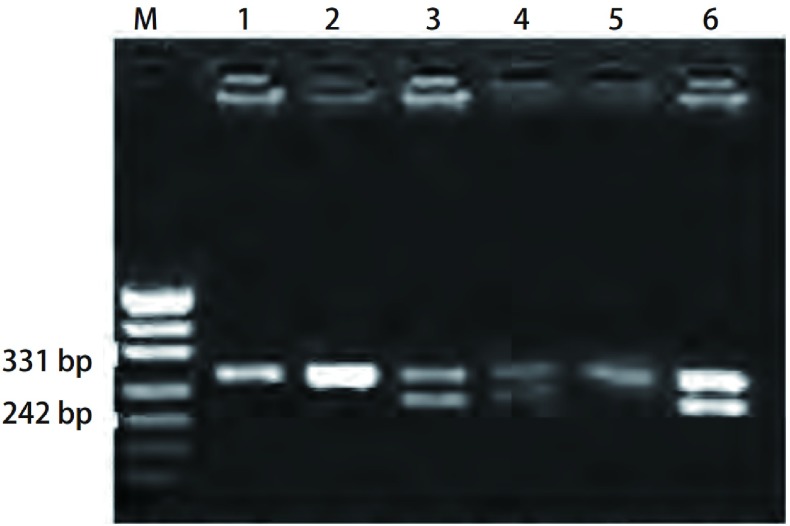
*GSTM1*基因分型。M：PUC19标志; 1、2、5泳道：缺陷型*GSTM1*（268 bp）; 3、4、6泳道：野生型*GSTM1*（268 bp和215 bp）。 *GSTM1* genotyping. M: PUC19 marker; 1, 2, 5 lines: null-*GSTM1* (268 bp); 3, 4, 6 lines: wide-type *GSTM1* (268 bp, 215 bp).

**2:**
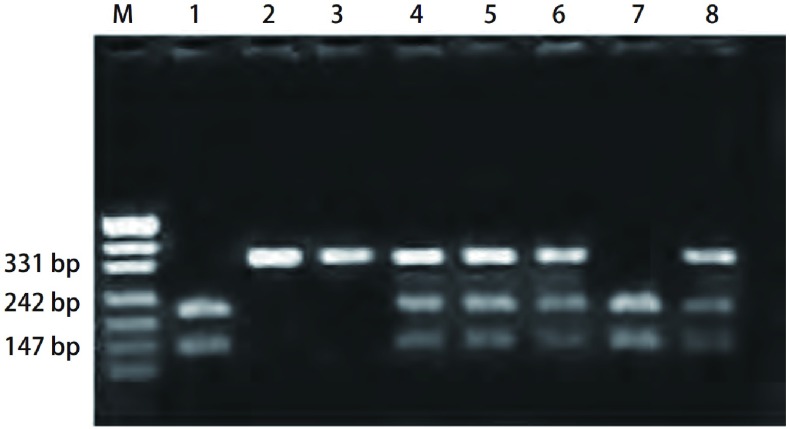
*CYP1A1*基因分型。M：PUC19标志; 2、3泳道：A型*CYP1A1*（340 bp）; 4、5、6、8泳道：B型*CYP1A1*（340 bp、240 bp和100 bp）; 1、7泳道：C型*CYP1A1*（240 bp和100 bp）。 *CYP1A1* genotyping. M: PUC19 marker; 2, 3 lines: the A type of *CYP1A1* (340 bp); 4, 5, 6, 8 lines: the B type of *CYP1A1* (340 bp, 240 bp, 100 bp); 1, 7 lines: the C type of *CYP1A1* (240 bp, 100 bp).

**3:**
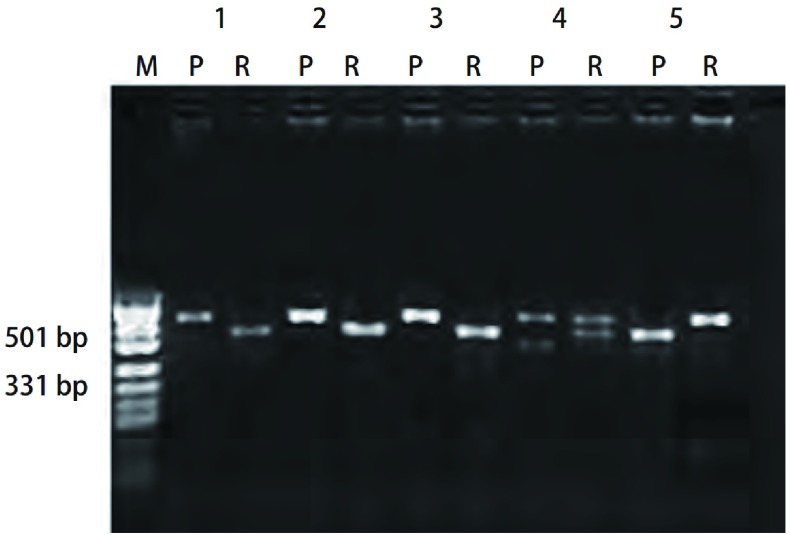
*CYP2E1*基因分型。M：PUC19标志; 1、2、3：A型*CYP2E1*[Rsa1(+/+)/Pst1(-/-)]，P泳道（410 bp），R泳道（360 bp）; 4：B型*CYP2E1* [Rsa1(+/-)/Pst1(+/-)]，P泳道（410 bp和290 bp），R泳道（410 bp和360 bp）; 5：C型*CYP2E1* [Rsa1(-/+)/Pst1(+/+)]，P泳道（290 bp和120 bp），R泳道（410 bp）。 *CYP2E1* genotyping. M: PUC19 marker; 1, 2, 3: the A type of *CYP2E1* [Rsa1(+/+)/Pst1(-/-)], P line (410 bp), R line (360 bp); 4: the B type of *CYP2E1* [Rsa1(+/-)/Pst1(+/-)], P line (410 bp, 290 bp), R line (410 bp, 360 bp); 5: the C type of *CYP2E1* [Rsa1(-/+)/Pst1(+/+)], P line (290 bp, 120 bp), R line (410 bp).

**4:**
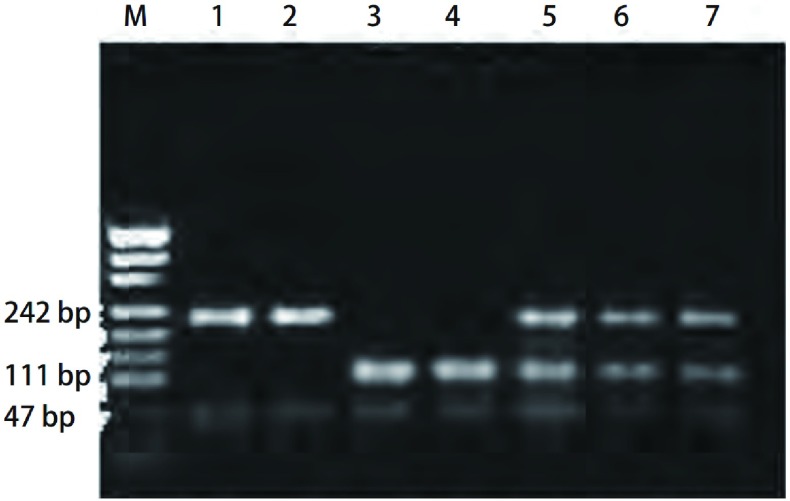
*CYP2D6*基因分型。M：PUC19标志; 1、2泳道：野生型*CYP2D6* (213 bp和59 bp); 3、4泳道：突变型*CYP2D6*（112 bp、101 bp和59 bp）; 5、6、7泳道：杂合型*CYP2D6*（213 bp、112 bp、101 bp和59 bp）。 *CYP2D6* genotyping. M: PUC19 marker; 1, 2 lines: the w/w type of *CYP2D6* (213 bp, 59 bp); 3, 4 lines: the m/m type *CYP2D6* (112 bp, 101 bp and 59 bp); 5, 6, 7 lines: the w/m type *CYP2D6* (213 bp, 112 bp, 101 bp and 59 bp).

### 1.4 化疗方案及疗效评价

全部患者的血常规、肝肾功能及体力状况评分等均具有化疗指征，化疗2周后进行疗效评价。参照RECIST标准判断疗效。对全部患者进行随访，最终随访日期为2011年1月，总生存期定义为患者诊断之日起至由于任何原因死亡或是末次随访时间。

### 1.5 统计学分析

采用SPSS 13.0统计学软件进行χ^2^检验、方差分析、分层分析及生存分析，*P* < 0.05为差异有统计学意义。

## 2 结果

### 2.1 四种药物代谢酶与化疗疗效的关系

#### 2.1.1 单一基因与化疗疗效的关系（表 3）

**3:** 四种药物代谢酶多态性与化疗疗效的关系 The relationship between the polymorphisms of 4 kind enzymes and chemotherapy effect

Genotyping		Chemotherapy		*X*^2^	*P*
		Response	No response		
*GSTM1*	Null	53	23	7.71	0.005^*^
	Non-null	23	28		
*CYP1A1*	A	37	14	5.206	0.023^*^
	B	29	26		
	C	10	11		
*CYP2E1*	A	41	28	0.661	0.719
	B	24	18		
	C	11	5		
*CYP2D6*	w/w	28	15	0.756	0.685
	w/m	23	17		
	m/m	25	19		
^*^P < 0.05.					

表 3提示缺陷型*GSTM1*的肺癌患者比功能型*GSTM1*的肺癌患者化疗疗效好（*P*=0.005），携带A型*CYP1A1*的肺癌患者比携带的B型和C型*CYP1A1*肺癌患者化疗疗效好（*P*=0.023），而*CYP2E1*和*CYP2D6*各基因型之间化疗疗效没有统计学差异（*P* > 0.05）。

#### 2.1.2 两种或两种以上基因与化疗疗效的关系

在两种或两种以上基因与化疗疗效关系的分析中仅*GSTM1*和*CYP1A1*有关联（表 4）。表 4提示同时携带缺陷型*GSTM1*和B型*CYP1A1*的肺癌患者比携带其它基因组合的肺癌患者化疗疗效好（*P* < 0.001）。

**4:** *GSTM1*和*CYP1A1*多态性与化疗疗效的关系 The relationship between the polymorphisms of *GSTM1*, *CYP1A1* and chemotherapy effect

*CYP1A1*	*GSTM1*	Chemotherapy effects		*X*^2^	*P*
		Response	No response		
A	Null	22	9	0.099	0.507
	Non-null	15	5		
B	Null	23	8	13.134	< 0.001^*^
	Non-null	6	18		
C	Null	8	6	^**^	0.361
	Non-null	2	5		
^*^P < 0.05; ^**^Fisher's exact test.					

#### 2.1.3 四种药物代谢酶与化疗方案的关系

表 5和表 6分别提示A型*CYP1A1*患者对非铂类化疗药物比B型和C型*CYP1A1*敏感，缺陷型*GSTM1*患者比功能型患者对铂类化疗药物敏感。

**5:** *CYP1A1*多态性与化疗方案的关系 The relationship of *CYP1A1* polymorphisms and chemotherapy programs

Chemotherapy programs	*CYP1A1*	Chemotherapy effects		*X*^2^	*P*
		Response	No response		
Platinum-drugs	A	12	5	1.632	0.442
	B	17	10		
	C	3	4		
Non-platinum-drugs	A	25	9	6.365	0.041^*^
	B	12	16		
	C	7	7		
^*^P < 0.05.					

**6:** *GSTM1*多态性与化疗方案的关系 The relationship of *GSTM1* polymorphisms and chemotherapy programs

Chemotherapy programs	*GSTM1*	Chemotherapy		*X*^2^	*P*
		Response	No response		
Platinum-drugs	Null	22	6	6.653	0.011^*^
	Non-null	10	13		
Non-platinum-drugs	Null	31	17	2.391	0.096
	Non-null	13	15		
^*^P < 0.05.					

### 2.2 四种代谢酶、化疗疗效、化疗方案等因素对肺癌患者生存的影响

#### 2.2.1 单因素对肺癌患者生存的影响

表 7及图 5显示在单因素分析中化疗疗效、化疗方案对肺癌患者生存有影响，化疗有效的肺癌患者比化疗无效的肺癌患者生存时间长，使用铂类化疗药物的肺癌患者比未使用铂类化疗药物肺癌患者生存时间短。而吸烟、病理分型、*GSTM1*、*CYP1A1*、*CYP2E1*和*CYP2D6*多态性对肺癌患者生存没有影响（*P* > 0.05）。

**7:** 肺癌患者单因素生存分析结果 The analysis of survival in the lung cancer patients

Factors		The median survival time (month)	The median survival time 95%CI (month)	*P*
Chemotherapy effects	Response	10	5.254-14.746	0.001^*^
	No response	5	3.251-6.749	
Chemotherapy programs	Platinum-drugs	5	1.501-8.499	0.011^*^
	Non-platinum-drugs	10	6.898-13.102	
^*^P < 0.05.				

**5:**
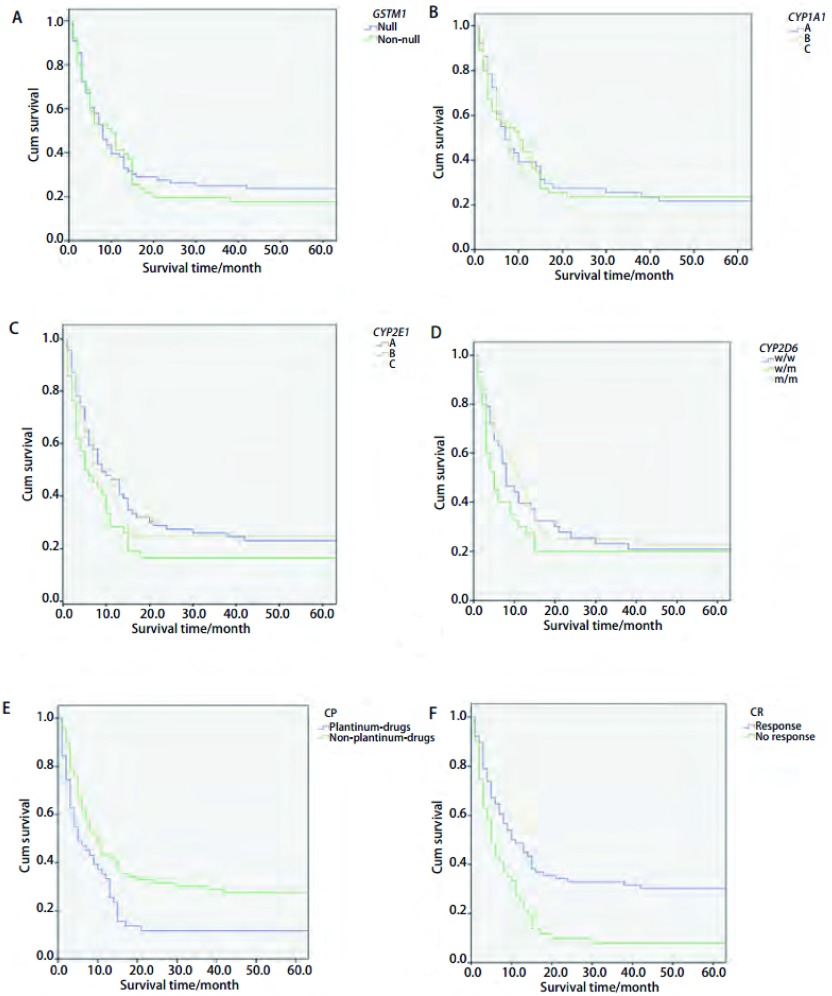
4种酶、化疗效果、化疗方案与患者生存之间的关系。A：*GSTM1*与患者生存之间关系; B：*CYP1A1*与患者生存之间关系; C：*CYP2E1*与患者生存之间关系; D：*CYP2D6*与患者生存之间关系; E：化疗方案与患者生存之间关系（CP：化疗方案）; F：化疗效果与患者生存之间关系（CR：化疗效果）。 The results of the association between 4 enzymes, chemotherapy response and chemotherapy programs and patients survival. A: the effect of *GSTM1* on the patients survival; B: the effect of *CYP1A1* on the patients survival; C: the effect of *CYP2E1* on the patients survival; D: the effect of *CYP2D6* on the patients survival; E: the effect of chemotherapy program on the patients survival (CP: chemotherapy programs); F: the effect of chemotherapy response on the patients survival (CR: chemotherapy response).

#### 2.2.2 多因素对肺癌患者生存的影响（表 8）

**8:** 对肺癌患者生存影响的多因素分析 Multivariate analyses of prognosis factors

Factors	*B*	Wald	*P*
Histological type	0.028	0.063	0.802
*GSTM1*	0.000	0.000	0.998
*CYP1A1*	-0.119	0.510	0.475
Smoking	-0.046	0.133	0.716
*CYP2E1*	0.01	0.003	0.954
*CYP2D6*	-0.213	2.138	0.144
Chemotherapy programs	-0.474	3.797	0.051
Chemotherapy effect	0.899	12.007	0.001^*^
^*^P < 0.05.			

由表 8可以看出在多因素分析中，*CYP2D6*、化疗方案、化疗疗效*P*值在0.05附近波动，为了得到最佳的回归模型、找到有意义的影响因素，我们按*P*值大小逐步对*CYP2D6*、化疗方案、化疗疗效3个变量进行进一步拟合，发现当模型中同时引入化疗方案和化疗疗效2个变量时，模型外其它变量不能再进入，模型内因子不能再剔除，由此我们得到最佳方程模型（表 9），筛选出对生存有显著影响的因素，即化疗方案和化疗疗效，该结果与单因素分析结果一致。

**9:** 化疗方案和化疗疗效对肺癌患者生存的影响 the effect of chemotherapy programs and chemotherapy effect on patients survival

Factors	B	Wald	*P*
Chemotherapy programs	-0.393	4.623	0.032^*^
Chemotherapy effect	0.548	8.691	0.003^*^
^*^P < 0.05.			

## 3 讨论

目前，国内外对Ⅰ、Ⅱ相代谢酶基因多态性与肿瘤化疗效果关系的研究已有相关报道，尤以国外为多，但多数研究集中在某一类酶中一种或多种多态性与肿瘤化疗关系的研究上，而且研究结果非常不一致^[6-8]^。GSTM1是Ⅱ相代谢酶，主要催化GSH与多环芳烃环氧化物共价结合，*GSTM1*基因多态性分为功能缺陷型和功能型; *CYP1A1*编码芳烃羟化酶，在其DNA结构的3’端PolyA下游碱基264T→C突变形成Msp1酶切点。CYP2E1参与亚硝胺及其前致癌物N-亚硝基二甲胺和N-亚硝基四吡咯烷的代谢，存在Rsa1和Pst1多态位点。CYP2D6ch型多态是由于在第一外显子上188C→T的突变，使第34位脯氨酸被丝氨酸取代，编码产生大量无活性酶。本研究我们检测研究对象*GSTM1*、*CYP1A1*、*CYP2E1*、*CYP2D6*四个多态位点，分析它们与病理分型、化疗药物、化疗效果之间的关系及与患者预后之间的关系。化疗有效组和无效组的年龄、性别没有统计学差异，排除了因年龄、性别分布不均衡可能造成对化疗效果的影响。

我们发现缺陷型*GSTM1*的肺癌患者比功能型肺癌患者化疗疗效好，尤其是携带缺陷型*GSTM1*和B型*CYP1A1*的肺癌患者，而*CYP2E1*和*CYP2D6*基因多态性与化疗疗效之间没有统计学差异。进一步分析发现携带A型*CYP1A1*肺癌患者接受非铂类化疗药物治疗比A型和C型患者疗效好，携带缺陷型*GSTM1*的肺癌患者接受铂类化疗药物治疗比功能型肺癌患者化疗疗效好。目前它们的多态性是否影响肺癌患者对化疗药物的应答以及对患者预后是否产生影响的研究结果不一致或很少报道，一些结果认为*GSTM1*缺陷型可能对化学药物治疗产生耐受^[9-11]^;也有研究^[12-15]^认为携带*GSTM1*缺陷型等位基因者对化疗敏感，这与我们的结果一致，这种研究结果的不一致可能是由于选择不同的药物代谢酶多态位点造成的，也可能是同一位点在不同肿瘤对化疗药物应答以及对肿瘤患者临床预后的影响存在差异。携带*GSTM1*缺陷型的肺癌患者对化疗敏感可能与其丢失了解毒的功能，使得化疗药物正常的发挥作用有关。至于携带A型*CYP1A1*肺癌患者接受非铂类化疗药物治疗比A型和C型患者疗效好，携带缺陷型*GSTM1*的肺癌患者接受铂类化疗药物治疗比功能型肺癌患者化疗疗效好的相关报道尚未见报道，这些提示可能对于临床用药有一定的帮助。

在单因素生存分析中化疗疗效和化疗方案对晚期NSCLC患者总生存期有影响，即化疗疗效好的患者比化疗疗效差的患者生存时间长，使用铂类化疗药物的肺癌患者比未使用铂类化疗药物肺癌患者生存时间短，该结论与临床的结论不太一致。本研究提示缺陷型*GSTM1*的肺癌患者比功能型的肺癌患者接受铂类化疗方案的化疗疗效好，但在76例携带缺陷型*GSTM1*中接受铂类化疗方案治疗的病例仅有28例（36.84%），其他48例患者接受了化疗疗效不好的非铂类药物治疗。而携带A型*CYP1A1*的肺癌患者对非铂类化疗方案比较敏感，在本研究中51例这类患者中接受非铂类化疗方案治疗的病例有34例（66.7%）。这种比例的不均衡可能造成了该结论与以往的结论不一致。而吸烟、病理分型、*GSTM1*、*CYP1A1*、*CYP2E1*和*CYP2D6*多态性对晚期NSCLC患者总生存期没有影响。

在*Cox*回归模型的拟合过程中发现当模型中同时引入化疗方案和化疗疗效2个变量时，模型外其它变量不能再进入，模型内因子不能再被剔除，得到了最佳方程模型，筛选出对生存有显著影响的因素，即化疗方案和化疗疗效，化疗方案和化疗疗效是影响晚期NSCLC的独立预后因素。*GSTM1*、*CYP1A1*、*CYP2E1*和*CYP2D6*多态性对晚期NSCLC患者总生存期没有影响。有研究^[16-, 17]^认为*GSTM1*是影响肺癌的独立预后因素，携带缺陷型*GSTM1*肺癌患者有更短的生存期，也有学者^[18, 19]^认为缺陷型*GSTM1*有助于改善患者的生存。对于*CYP2D6*多态性与化疗疗效以及对肿瘤患者预后影响的研究较多，但由于研究的多态位点不同所以得到的结论也不尽相同^[20-25]^。关于*CYP1A1*、*CYP2E1*多态性与化疗疗效以及对肿瘤患者预后影响的研究较少。

总之，缺陷型*GSTM1*的肺癌患者比携带功能型*GSTM1*的肺癌患者化疗疗效好，尤其是对以铂类为基础的化疗方案。尽管*GSTM1*和*CYP1A1*多态性对晚期NSCLC患者总生存期没有影响，但是携带缺陷型*GSTM1*肺癌患者对以铂类为基础的化疗方案相对敏感，因此这部分患者将可能从铂类为基础的化疗方案中获益; 而A型*CYP1A1*肺癌患者对以非铂类为基础的化疗方案相对敏感，因此这部分患者将可能从非铂类为基础的化疗方案中获益。
